# Biphasic Pulmonary Blastoma: A Rare Tumor With Fatal Outcome—First Reported Case From Lebanon

**DOI:** 10.1002/ccr3.72325

**Published:** 2026-03-19

**Authors:** Houssein Chahrour, Georges Nammour, Joseph A. Akiki, Mohammad H. Abbas, Wassim Hamadeh

**Affiliations:** ^1^ School of Medicine University College Cork Ireland; ^2^ School of Medicine and Medical Sciences Holy Spirit University of Kaslik Jounieh Lebanon; ^3^ Faculty of Medicine Saint Joseph University Beirut Lebanon; ^4^ Faculty of Medical Sciences Lebanese University Beirut Lebanon; ^5^ National Institute of Public Health Clinical Epidemiology and Toxicology–Lebanon (INSPECT‐LB) Beirut Lebanon

**Keywords:** biphasic pulmonary blastoma, lung cancer, oncology, pulmonary blastoma, rare tumors

## Abstract

Biphasic pulmonary blastoma is a rare lung malignancy, accounting for less than 0.5% of all primary lung tumors. It is characterized by a biphasic nature of those tumors that consist of an admixture of epithelial and mesenchymal components. Up to this date, no standardized treatment approach has been developed and patient's prognosis remains poor. We present the case of an 85 year‐old female who presented with dyspnea, associated with cough and hemoptysis, that has rapidly evolved over the past weeks, along with severe weight loss and anorexia. The patient was ultimately diagnosed with biphasic pulmonary blastoma on a liver core biopsy. To the best of our knowledge, this is the first case of biphasic pulmonary blastoma to be reported in Lebanon. The aggressive biological behavior of this rare tumor is highlighted by the occurrence of liver metastasis early on. It underlines the importance of recognizing this entity, even in metastatic sites. Death occurring within days of biopsy illustrates the fulminant course of those malignancies and its capacity to outpace routine diagnostic evaluation.

## Key Clinical Message

Biphasic pulmonary blastoma is a highly aggressive malignancy with early distant metastasis that may be mistaken for metastatic disease originating from an extrapulmonary primary tumor. It should be considered in the differential diagnosis of large, rapidly growing pulmonary masses.

## 1 Introduction

Pulmonary blastoma (PB) was first described in 1945 by Barrett and Barnard as an “embryoma of the lung” due to its resemblance to fetal lung tissue, hence the term “blastoma,” emphasizing its primitive features. It was later named “carcinosarcoma” by Spencer [1]. Subsequent studies refined its classification based on age distribution, biological behavior, and stromal malignancy. Currently, pulmonary blastoma comprises three distinct categories: (1) well‐differentiated fetal adenocarcinoma (WDFA), composed solely of epithelial elements; (2) pleuropulmonary blastoma (PPB), composed exclusively of mesenchymal tissues; and (3) classic biphasic pulmonary blastoma (CBPB), which is the only biphasic category, characterized by a histological admixture of epithelial and mesenchymal malignancies [2, 3, 4].

Herein, we report the case of an 85‐year‐old female diagnosed with metastatic biphasic pulmonary blastoma, highlighting the clinical presentation, diagnostic challenges, and aggressive course of this rare entity.

## 2 Case Presentation

**FIGURE 1:**
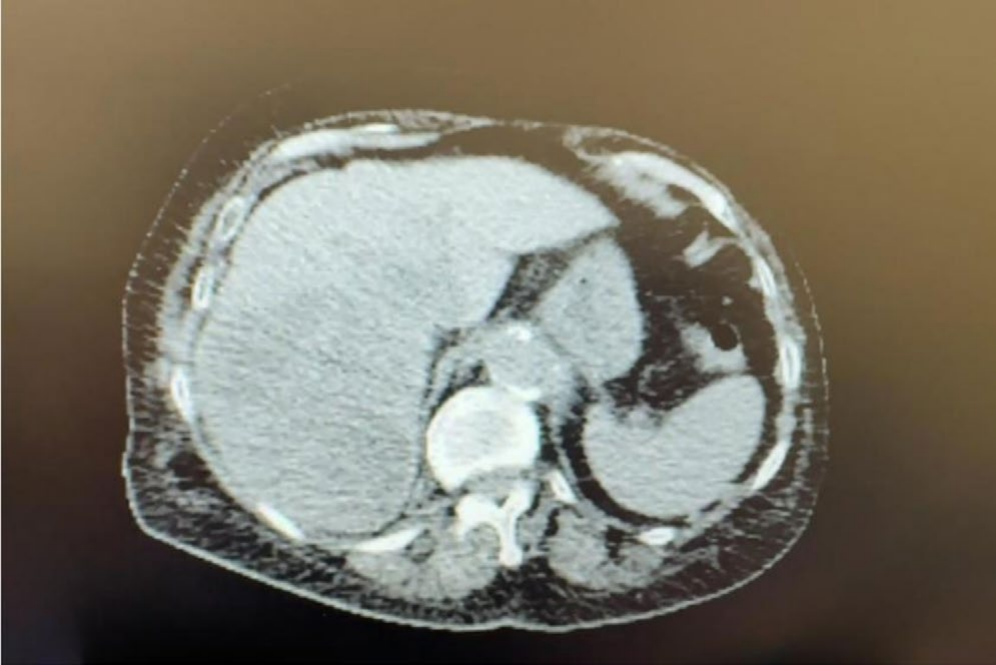
Computed tomography revealed the presence of necrotic liver lesions.

An 85‐year‐old Iraqi woman presented to our clinic complaining of severe dyspnea at rest, which had been relatively evolving over the past few weeks. The patients medical history is relevant to diabetes mellitus type 2 and hypertension, both adequately managed with appropriate medical therapy. She also reported a cough associated with episodes of hemoptysis and had consulted multiple physicians prior to presentation. On physical exam, the patient appeared cachectic, pale, and fatigued. Pulmonary auscultation revealed markedly decreased air entry over the left hemithorax. Abdominal examination revealed a slightly enlarged, non‐tender liver. Given the high clinical suspicion for lung malignancy, the patient was admitted for further evaluation. Laboratory test results are shown in Table 1. They were most significant for a normocytic anemia along with leukocytosis dominated by neutrophils and lymphopenia. For staging and disease assessment, a computed tomography (CT) scan was initially performed, followed by positron emission tomography (PET). The PET/CT revealed a radiotracer‐avid pleural mass in the posterior segment of the left lower lobe, measuring 4.1 × 3.9 × 5.1 cm, with a maximum standardized uptake value (SUVmax) of 13.5. An additional radiotracer‐avid nodule was identified in the anterior segment of the right upper lobe, measuring 1.2 cm, with an SUVmax of 2.8. Furthermore, three radiotracer‐avid hypodense necrotic hepatic lesions were detected in segments VIII, VII, and VI, measuring approximately 14.3 × 11.1 × 12.3 cm, with SUVmax of 10 (Figure 1).

**TABLE 1:** Complete blood count results showing normocytic anemia along with leukocytosis dominated by neutrophils and lymphopenia.

Test	Result	Unit	Reference Range
WBC	**13.23 (High)**	10^3^/μL	4.5–10.5
Segmented Neutrophils	**86.8 (High)**	%	25–65
Lymphocytes	**7.3 (Low)**	%	15–40
Monocytes	5.2	%	1–6
Eosinophils	**0.2 (Low)**	%	0.6–4
Basophils	0.5	%	0–1
Hemoglobin	**9.5 (Low)**	g/dL	12–18
Hematocrit	**29.2 (Low)**	%	37–52
RBC	**3.45 (Low)**	10^6^/μL	4–5.5
MCV	84.6	fL	78–97
MCH	27.5	pg	26–33
MCHC	32.5	g/dL	30–35
RDW‐CV	14.6	%	11.6–14.6
Platelets Count	445	10^3^/μL	150–450

At that stage, hepatocellular carcinoma and metastatic lung tumor were among the differential diagnoses. Histopathological assessment was obtained through a liver biopsy using a needle. Unfortunately, the patient passed away before a definitive diagnosis was established.

**FIGURE 2:**
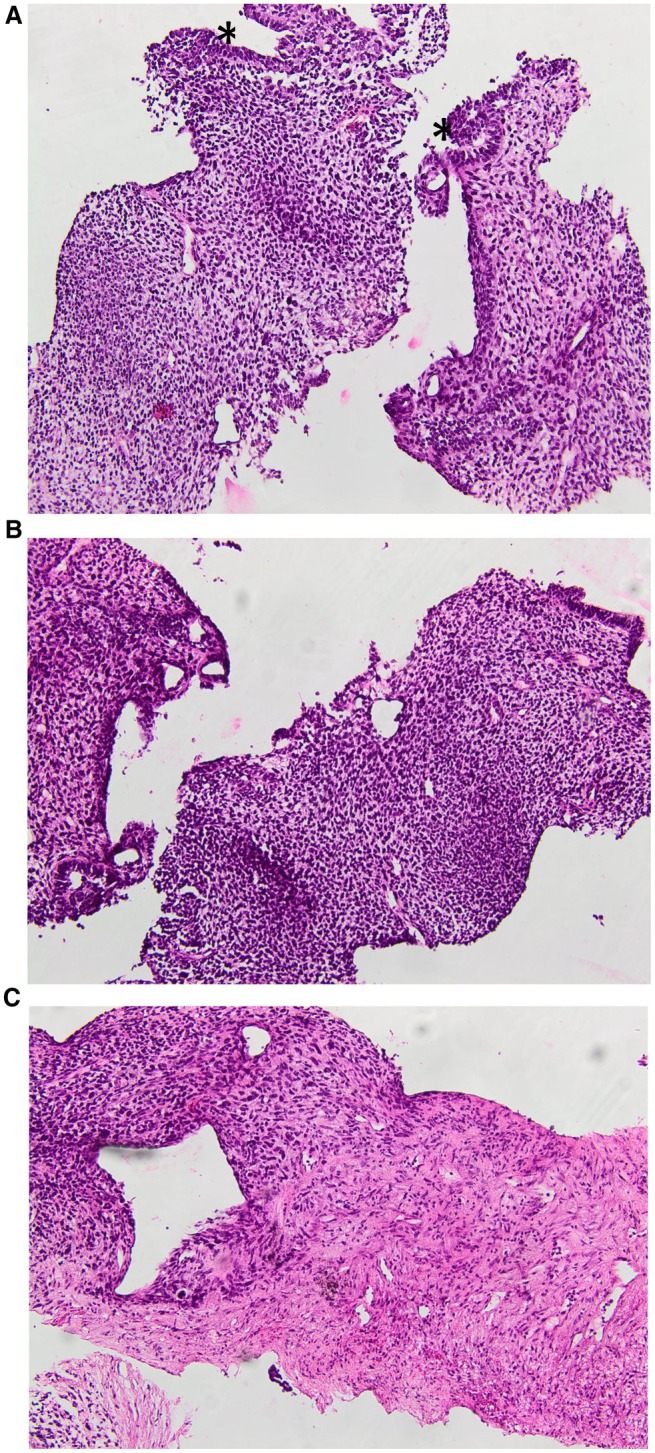
(A) biphasic aspect of the tumor consisting of a glandular epithelium and a blastematous stroma. The glandular structures (*) are lined by pseudostratified columnar cells showing minimal nuclear atypia (H&E, LP). (B) Biphasic aspect of the tumor consisting of a glandular component and a blastematous stroma (H&E, LP). (C) Primitive mesenchymal component was made up of closely packed primitive, oval to spindle‐shaped cells. There were no foci of specific mesenchymal differentiation (H&E, LP).

**FIGURE 3:**
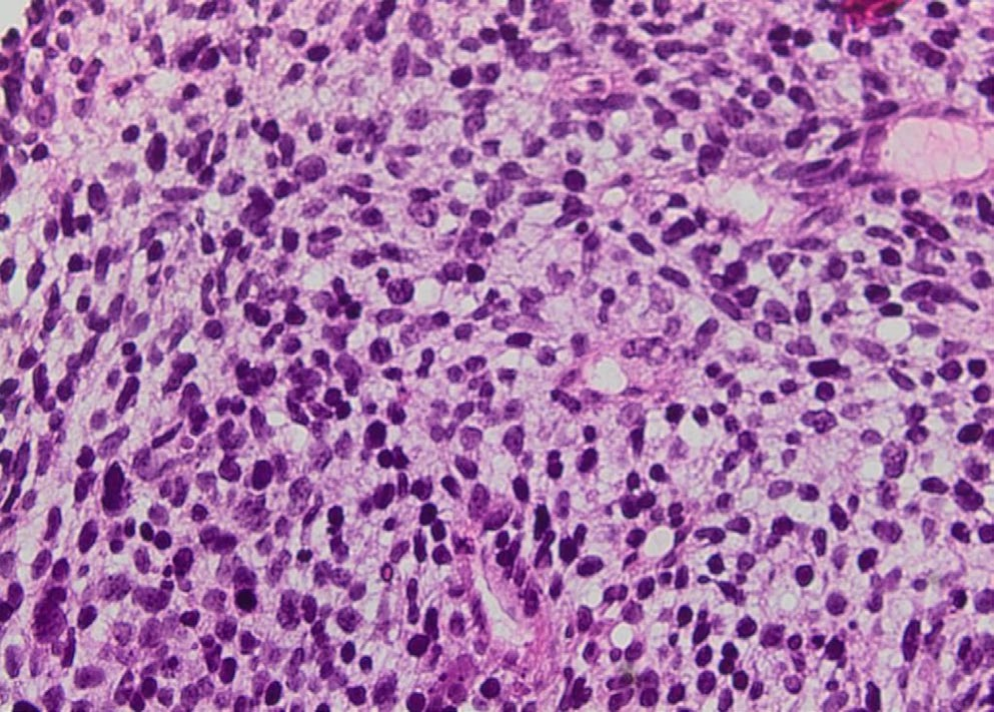
On high power, epithelial cells had small, round to oval nuclei and clear to slightly cytoplasm containing glycogen vacuoles.

**FIGURE 4:**
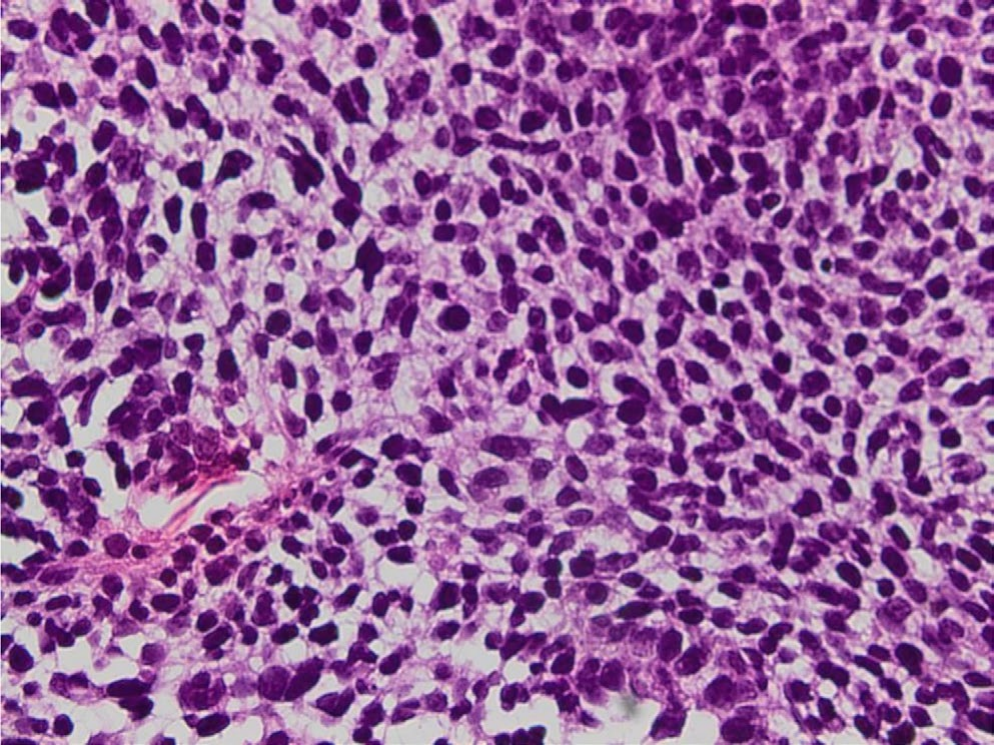
Epithelial cells on high power have small, round to oval nuclei and clear to slightly cytoplasm containing glycogen vacuoles.

Paraffin block sections from the liver metastasis demonstrated a poorly differentiated component. The latter was composed of a glandular component and a primitive blastematous mesenchymal component (Figures 1 and 2A–C). The glandular component consisted of a pseudostratified epithelium and a branched glandular structure (Figures 1 and 2A–C). The cells showed vesicular nuclei with minimal atypia (Figures 3 and 4). The mesenchymal component was predominantly undifferentiated, composed of closely packed oval to spindle‐shaped cells. No heterologous mesenchymal differentiation was noted on light microscopy, including any osteosarcomatous, chondrosarcomatous, or rhabdomyosarcomatous differentiation.

**FIGURE 5:**
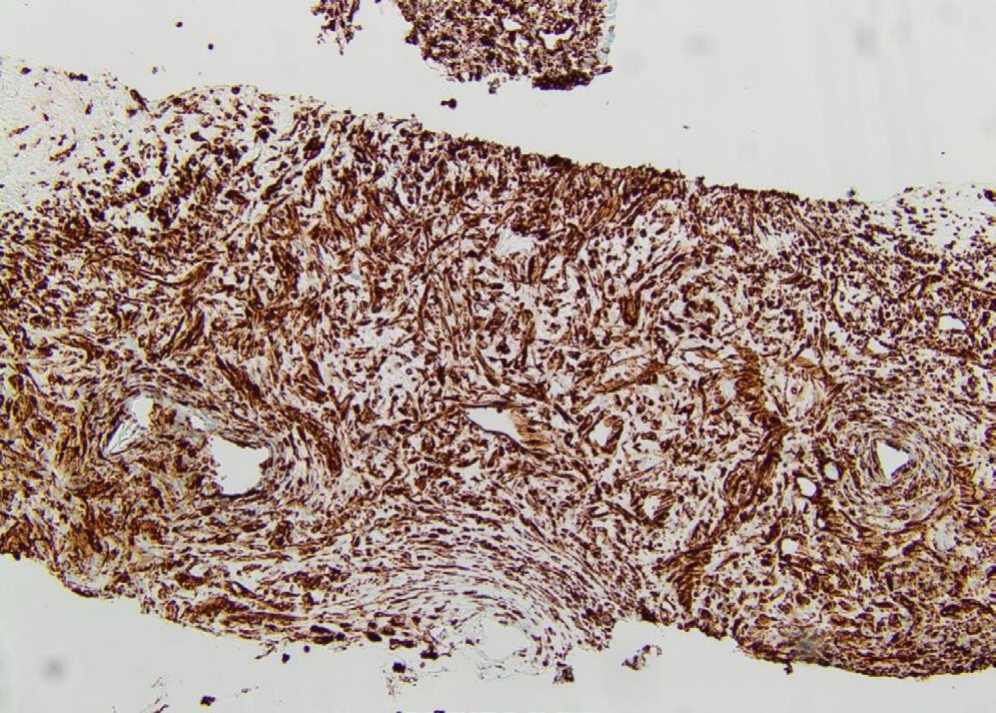
Vimentin immunostaining is positive in the mesenchymal component.

**FIGURE 6:**
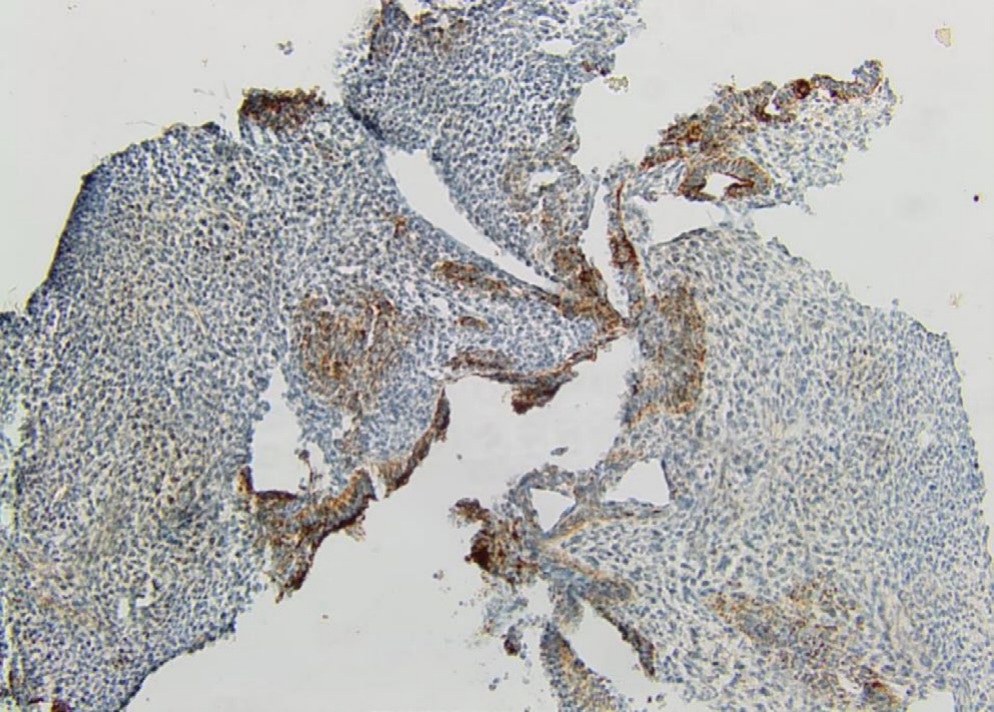
Cytokeratin immunostaining highlighting the glandular component.

On Immunohistochemistry, the mesenchymal component was positive for Vimentin (Figure 5) and the glandular component showed positivity for cytokeratin stain (Figure 6). The biphasic nature of the tumor was therefore established.

## 3 Differential Diagnosis

Fetal‐type adenocarcinoma, pleuropulmonary blastoma, biphasic sarcomas such as biphasic‐type synovial sarcoma, and metastatic tumors—especially malignant Mullerian tumors from the gynecological tract—were among the primary differential diagnoses [2]. However, there was no blastematous component as in fetal‐type adenocarcinoma. Pleuropulmonary blastoma occurs typically in children and has a peripheral location and a cystic appearance. Almost all synovial sarcomas have the X:18 SS18‐SSX translocation, are monophasic, and do not have a glandular component [4]. In our case, a molecular study for this specific translocation was not made due to lack of resources. There is no fetal adenocarcinoma morphology in the glandular component of biphasic synovial sarcomas. A high‐grade fetal adenocarcinoma may represent the epithelial component of carcinosarcomas, although this should be differentiated from pulmonary blastoma. In these situations, β catenin is only found in the epithelial membrane. The identification of metastatic mixed Müllerian tumors is aided by a history of uterine tumors. Immunohistochemistry for hormonal receptors could be useful in this case [2, 4].

Based on the characteristic biphasic morphology, histomorphological features, and immunophenotypic profile, and in correlation in conjunction with the patient's clinical and radiological findings, the diagnosis of metastatic pulmonary blastoma was established.

## 4 Discussion

Biphasic pulmonary blastoma accounts for approximately 0.25%–0.5% of all primary lung malignancies [3]. Histopathologically, it is defined by areas of resemblance to the embryonal lung during the pseudoglandular stage of lung development, which occurs between [3, 4]. The tumor is biphasic and composed of an admixture of two components: an epithelial glandular component that recapitulates features of fetal lung development, and the other is stromal mesenchymal. The latter is made up of primitive spindle or oval cells. Heterologous differentiation, most commonly rhabdomyosarcomatous or chondrosarcomatous, is reported in around 25%–30% of cases and is associated with a poorer prognosis [5, 6]. Extensive necrosis and hemorrhage are characteristic of rapidly proliferating tumors that outgrow their vascular supply [5]. Diagnosis of BPB relies on immunohistochemical profiling. The epithelial component is usually positive for CK7, AE1/AE3, EMA, and TTF‐1 (positive in about 85% of cases), confirming pulmonary origin [7]. The mesenchymal component expresses vimentin and lacks cytokeratin expression [3]. Diffuse nuclear β‐catenin staining is a defining diagnostic feature and reliably distinguishes BPB from morphologic mimics [8]. However, as β‐catenin immunostaining is a specialized immunohistochemical technique used to assess activation of the Wnt/β‐catenin signaling pathway, it is not part of routine immunohistochemical panels and is typically performed when specific tumors are suspected. High CD117 (c‐kit) expression in both components further supports a monoclonal stem cell origin [3, 4].

Epidemiologic studies indicate that classic biphasic pulmonary blastoma is most commonly diagnosed during the fourth and fifth decades of life. As certain series report a male‐to‐female ratio of approximately 2:1 and a smoking history present in more than the vast majority, there seems a predilection for smoking male patients.

Patients can come often to clinical attention with nonspecific complaints or can even be symptomatic presentations. They can include, for instance, pulmonary complaints such as persistent cough, hemoptysis, or, as in our case, dyspnea, all of which can often mimic more common lung diseases. Additionally, symptoms related to mass effect may occur. Systemic and constitutional symptoms such as weight loss, fatigue, and anorexia are not uncommon.

These tumors typically present as peripheral, unencapsulated, and solitary lung masses, with a reported predilection to the upper lobes. They show up radiologically as a large, well‐defined peripheral pulmonary nodule or mass [5]. On CT, they have heterogeneous density, with areas of low density suggestive of the necrotic areas, and heterogeneous enhancement of the soft tissue. The FDG uptake on PET‐CT demonstrated markedly increased metabolic activity, suggesting aggressive disease with a high metabolic rate [2, 4, 5]. On the other hand, non‐specific imaging features such as intratumoral septations, intratumoral hemorrhage, and cystic change can be seen on Magnetic Resonance Imaging [2].

BPB is a highly lethal tumor because of its dual nature of containing both a low‐grade fetal adenocarcinoma and a malignant mesenchymal stromal component [9]. While the epithelial component generally metastasizes through the lymphatic route, the mesenchymal component metastasizes through the hematogenous route. This allows the tumor to have a dual metastatic spread [4, 5]. Cases reported in autopsy series mention typical sites of early metastatic spread as the brain, bone, liver, and adrenal glands, although radiologically occult on initial presentations [6]. This combined spread likely explains the rapid failure observed in most cases.

Molecular studies indicate that BPB is monoclonal, originating from a single pluripotent stem cell [3, 10]. This is also confirmed by allelic imbalance, showing genomic alterations involving chromosomal regions 14q24–q32 and 17p11–p13 [10]. A key driver is aberrant activation of the Wnt/β‐catenin pathway, with CTNNB1 exon 3 mutations identified in 71%–86% of BPB cases [9]. These mutations prevent β‐catenin degradation, leading to nuclear accumulation and transcriptional activation of Wnt target genes that maintain an undifferentiated, fetal‐like phenotype [11]. This is reflected in the strong nuclear β‐catenin staining seen in squamoid morules [3].

In our case, on immunohistochemistry (Table 2), the glandular component is reactive to cytokeratin and carcinoembryogenic antigen, while the stromal component shows positivity for vimentin and SMA. Both components show positivity for β catenin [9]. Some cases might show rhabomyoblastic differentiation, which can be highlighted by myogenin, MyoD1, and desmin. However, this remains a non‐consistent finding. S100 and vimentin are helpful markers in rare cases, showing fibroblasts that are arranged in bands or nodules in association with nodules of cartilage. The Ki‐67 proliferation index shows variable expression in the mesenchymal layer [12].

**TABLE 2:** Immunohistochemical and Genetic Features of Biphasic Pulmonary Blastoma.

Immunohistochemical markers	Genetic aberrations
*Glandular component:* CKs, CEA, β catenin	8q gains (sporadic) Trisomy 2 17p deletions
*Stromal component:* SMA, vimentin, β catenin *If cartilaginous or rhabdomyoblastic differenciations:* S100, Myogenin, myoD1	DICER1 (familial)

Abbreviations: CEA, carcinoembryogenic antigen; CKs, Cytokeratins; SMA, smooth muscle actin.

Molecularly, familial PPB has been shown to harbor germline abnormalities in the DICER1 gene located on chromosome 14q32. In sporadic cases, gains of chromosome 8q have been found as a recurring event, while trisomy 2 and 17p deletions were rarely reported [13]. DICER1 mutations are present in around 80%–90% of BPB cases [9, 14]. These typically follow a two‐hit mechanism involving a truncating germline or somatic mutation combined with a somatic RNase IIIb missense mutation, resulting in defective 5p miRNA processing and dysregulated developmental gene expression [9]. More recent sequencing analyses have also pointed out the involvement of TP53, KRAS, MET amplification, ROS1 rearrangements, EGFR mutations, BRCA2 deletion, and MYCN amplification, though not in a consensual manner and lacking obvious therapeutic implications at this juncture [3, 15]. Epigenetic abnormalities also play their due role in tumorigenesis. The deletion of the locus of imprinting at the IGF2 locus introduces bi‐allelic expression and activation of the downstream signaling of PI3K/AKT/MRT and MAPK pathways due to the lack of control on proliferation [9, 16].

Given the rarity of those tumors, a clear or standardized approach to treatment has yet to be developed. Up to this day, most clinical decisions are guided by what has been learned from individual case reports or small retrospective reviews. Surgery, however, remains the mainstay, and when a complete resection with clear margins can be achieved, it offers the best hope for longer survival [4, 5, 17]. Unfortunately, even with successful surgery, recurrence is common and usually develops within the first two years [4, 17]. Adjuvant chemotherapy or radiotherapy is sometimes used, especially in patients with incomplete resection, metastatic spread, or more aggressive histologic features, but outcomes have been mixed [4, 17]. The most frequently used regimens involve cisplatin or carboplatin, combined with etoposide, vincristine, or ifosfamide [4]. A few reports describe short‐lived responses to doxorubicin and ifosfamide, but these benefits are rarely sustained [4]. Furthermore, changes in CTNNB1, DICER1, and MET have been reported for future targeted therapies, though none have yet shown clinical benefit [3, 4, 9]. Overall, despite surgical resection remaining the cornerstone of treatment, the prognosis of BPB remains poor, with 5‐year survival rates around 15%–16%, and median survival of approximately 1 year [4]. The few long‐term survivors described in the literature generally had smaller, localized tumors that could be completely removed at diagnosis [4, 5, 17]. Historical series show poor long‐term survival, with older literature suggesting ~16% 5‐year survival, two‐thirds of patients dying within 2 years, and recurrence frequently within the first year after surgery [15, 17].

## 5 Limitations

This report is limited by the absence of comprehensive molecular testing, including β‐catenin immunostaining and DICER1 mutation analysis, which could not be performed due to local resource constraints. Nevertheless, the diagnosis was supported by the characteristic biphasic morphology, immunohistochemical profile, and clinic‐radiological correlation. Recognizing biphasic pulmonary blastoma in the Lebanese population is essential to raise clinical awareness of this rare and highly aggressive malignancy and to prevent misdiagnosis in patients presenting with rapidly progressive pulmonary masses.

## 6 Conclusion

Biphasic pulmonary blastoma is an exceptionally rare and highly aggressive malignancy that poses significant diagnostic challenges due to its rapid progression and early metastatic spread. Awareness of this entity is essential to avoid misdiagnosis and to facilitate timely management. Additional case reports and case series are necessary to gain deeper insights into their clinical behavior, diagnostic challenges, and optimal management strategies.

## Author Contributions

**Houssein Chahrour:** conceptualization, investigation, methodology, validation, writing – original draft, writing – review and editing. **Georges Nammour:** investigation, methodology, validation, visualization, writing – original draft, writing – review and editing. **Joseph A. Akiki:** investigation, methodology, validation, writing – review and editing. **Mohammad H. Abbas:** investigation, methodology. **Wassim Hamadeh:** conceptualization, investigation, supervision, visualization, writing – original draft, writing – review and editing.

## Funding

The authors have nothing to report.

## Ethics Statement

Ethical approval for publication of this case report was obtained from the Lebanese University in June 2025, ref. 8/2025.

## Consent

Written informed consent for publication of this case report and any accompanying images were obtained from the patient's next of kin.

## Conflicts of Interest

The authors declare no conflicts of interest.

## Data Availability

The authors have nothing to report.
